# Effectiveness of Group-Delivered Cognitive Therapy and Treatment Length in Women Veterans with PTSD

**DOI:** 10.3390/bs4010031

**Published:** 2014-01-10

**Authors:** Diane T. Castillo, Katharine Lacefield, Janet C’de Baca, Abby Blankenship, Clifford Qualls

**Affiliations:** 1 Behavioral Health Care Line, New Mexico VA Health Care System, 1501 San Pedro SE, Albuquerque, NM 87108, USA; E-Mail: janet.cdebaca@va.gov; 2 Department of Psychiatry, School of Medicine, MSC 09 5030, University of New Mexico, 1 University of New Mexico, Albuquerque, NM 87131, USA; 3 Psychology Department, Washington DC VA Medical Center, 50 Irving Street NW, Washington, DC 20422, USA; E-Mail: Katharine.lacefield@va.gov; 4 Department of Psychiatry, School of Medicine, University of Texas Health Sciences Center at San Antonio, 7550 IH 10 West, Suite 1325, San Antonio, TX 78229, USA; E-Mail: abby.e.blankenship@gmail.com; 5 Clinical & Translational Science Center (CTSC), University of New Mexico, MSC 09 5030, University of New Mexico, 1 University of New Mexico, Albuquerque, NM 87131, USA; E-Mail: cqualls@salud.unm.edu

**Keywords:** PTSD, cognitive processing therapy, group therapy, women veterans

## Abstract

The effectiveness and length of group-delivered cognitive treatment for Posttraumatic Stress Disorder (PTSD) was examined in a sample of women veterans. The sample included 271 primarily non-Hispanic white (61%) and Hispanic (25%) women veterans treated in 8-, 10-, or 12-group length sessions with manualized cognitive therapy for PTSD. Outcome was measured with the PTSD Symptom Checklist (PCL) in an intention-to-treat analysis (*N* = 271), in completer subjects (*n* = 172), and with group as the unit of analysis (*n* = 47 groups). Significant decreases in PTSD were found in the full sample (effect size [*ES*] range = 0.27 to 0.38), completers (*ES* range = 0.37 to 0.54), and group as the unit of analysis (*ES* range = 0.71 to 0.92), suggesting effectiveness of cognitive group treatment for PTSD. PCL scores significantly improved in the 8, 10, and 12 group lengths, with no differences between each. Clinical improvement showed a third decreasing 10 or more PCL points and 22% no longer meeting PTSD diagnostic criteria, with the best results in the 10-session group. The results suggest group-delivered cognitive therapy is an effective, efficient, time-limited treatment for PTSD.

## 1. Introduction

Cognitive Processing Therapy (CPT) [1] has been established as a standard of care for the treatment of Posttraumatic Stress Disorder (PTSD) [2], recommended as a first line of treatment [3], and nationally disseminated to PTSD therapists in the Department of Veterans Affairs (VA) [4]. CPT is a manualized cognitive-behavioral treatment intervention, founded in emotional processing theory [5], which suggests PTSD develops due to an interruption in the natural recovery process after the experience of a traumatic event. The focus of CPT is on accessing and modifying altered cognitive structures [6], rather than direct emotional processing utilized in Prolonged Exposure therapy [5]. The 12-session protocol applied to veterans [7] includes education and application of cognitive restructuring principles to assimilated and over-accommodated beliefs regarding self, others, and world perceptions; modification of these beliefs in five domains—safety, trust, power/control, esteem, and intimacy—and some trauma processing. Altered cognitions in the five domains after a traumatic event disrupt functioning and result in PTSD symptoms.

The CPT protocol provided individually has demonstrated efficacy, showing relevant within-treatment effect sizes (range = 0.92 to 2.75) [2] in numerous studies across trauma types, including combat, natural disaster, and interpersonal violence [8,9]; and combat eras, including Vietnam and Operation Enduring Freedom/Operation Iraqi Freedom [10]. In addition to statistically significant post-treatment reductions in PTSD, clinically significant symptom reduction is maintained 5–10 years after treatment [11], suggesting long-term improvement in PTSD with CPT. It should be noted that prior to the development of the manualized CPT protocol, other cognitive therapy interventions also demonstrated efficacy in individual trials with comparably large effect sizes (range = 1.10 to 2.46) [2]. Delivery of CPT therapy has also been narrowed to exclude the Trauma Account component, with focus exclusively on cognitive skills (CPT-C) and has demonstrated equivalent within-treatment effect sizes (*ES* = 1.45) [12].

The evidence for individually delivered CPT and cognitive restructuring is well-established for the treatment of PTSD and, although the components of CPT can be readily applied in a group setting, the literature supporting group delivery of CPT, as with general cognitive restructuring treatment interventions, is limited. Cognitive restructuring treatments are conceptually considered under the rubric of cognitive-behavioral interventions, and most group examinations have included a variety of both cognitive and behavioral treatments, making attributions to the cognitive component alone problematic. Among the 16 randomized controlled cognitive-behavioral group trials in a recent meta-analysis [13], none examined CPT or cognitive restructuring separate from other interventions such as exposure, anger management, seeking safety, and others. While Chard [14] examined CPT in a group setting, the group sessions were alternated with individual, limiting conclusions on the group contribution to the treatment results. Resick and Schnicke [6] reported positive effects with CPT in group (*ES* = 0.89) in a non-randomized study, and two other more recent non-randomized trials also found support for group CPT. Zappert and Westrup [15] describe significant within-treatment PTSD reductions for survivors of military sexual trauma in a residential program using a modified group CPT protocol; however, conclusions on the efficacy of group CPT were confounded by other treatment interventions provided in the residential program. In another residential program study [16], group CPT produced significant reductions in PTSD and depressive symptomatology compared to a treatment-as-usual group. In addition, individuals who received CPT exhibited significant improvements in quality of life, coping, and psychological distress, and were more likely to be identified as recovered at discharge. While the findings of the latter study are important in suggesting effectiveness of CPT in a group when compared to an active control, of concern was the potential of other interventions influencing the results in the residential program and the retrospective nature of the study. 

Although the group literature for cognitive restructuring and CPT is not comparable to the individual literature, the consideration of group as a modification in the delivery of CPT is reasonable, as both Sloan *et al.* [13] and a review of the literature on cognitive-behavioral group interventions for PTSD [17] determined efficacy for the group delivery of PTSD treatments. If efficacy and effectiveness can be established for a group CPT intervention, other benefits arise with group, such as those outlined by Yalom [18] in curative factors (e.g., universality, catharsis, *etc.*), as well as efficiency and increased access in the delivery of treatment. Thus, modifying and systematically examining the factors that contribute to the improvement of PTSD in altering aspects of the standard-of-care protocol will help to better understand the necessary and sufficient factors that contribute to PTSD change using cognitive restructuring treatments. 

This study examined the application of the group delivery cognitive therapy with three varying treatment lengths in a clinical outpatient sample of women veterans. The first aim was to combine and examine treatment improvement in PTSD for group-delivered cognitive treatment. The second aim was to compare the three cognitive treatment lengths delivered in group: a 12-session CPT-C protocol, a 10-session modified CPT-C treatment protocol, and a further modified 8-session cognitive therapy variation. Our hypotheses were: 1) the combined group-delivered cognitive treatment variations will reduce PTSD symptomatology, and 2) a greater number of group sessions will produce greater PTSD change.

## 2. Method

### 2.1. Participants

Data were collected from 271 women veterans treated for PTSD in 51 groups between 1995 and 2013 in a Southwest VA women’s trauma outpatient clinic. PTSD was determined by psychological testing and structured interviews. All data were collected through archival record review and approved by local VA and University of New Mexico Institutional Review Board. Demographics for the total study sample and by through group lengths are provided in Table 1. The average age was 45.0 (*SD* = 10.2); participants were primarily non-Hispanic white (61%) or Hispanic (25%); most experienced more than one trauma (82%) in both childhood and adulthood (56%), and sexual traumas were the most frequently reported, with 53% sexual trauma alone and 43% sexual trauma with another trauma type; 67% were diagnosed with a co-morbid psychiatric diagnosis in addition to PTSD. Completers (*n* = 172) were defined as participants not missing post-treatment PCL scores and were compared to non-completers (*n* = 99) on demographic characteristics and CAPS scores. Completers were significantly older (*M* = 46.03, *SD* = 9.76) than non-completers (*M* = 43.31, *SD* = 10.76) at entering treatment (*p* = 0.04) and had fewer number of traumas (completers: number of traumas ≥ 2) than non-completers (number of traumas = 1; *p* = 0.03), with no other significant differences. The characteristics of the 172 completer subjects reflected entry level PTSD scores on the CAPS similar to other clinical populations (Current: *M* = 75.8, Lifetime: *M* = 107.9, Total: *M* = 182.1) [19]. 

**Table 1:** Demographics, trauma characteristics, and CAPS scores for total sample and within groups.

	Total Sample *N* =271	8-Session Ind *n* = 117† Group *n* = 32‡	10-Session Ind *n* = 17† Group *n* = 5‡	12-Session Ind *n* = 38† Group *n* = 9‡
**Number of Members/group**		*M* = 7.72 (range: 3 to 14)	*M* = 6.20 (range: 4 to 8)	*M* = 7.56 (range: 5 to 11)
	*M (SD)*	*M (SD)*	*M (SD)*	*M (SD)*
**Age**	45.0 (10.2)	45.8 (9.7)	47.4 (11.2)	46.1 (9.5)
**CAPS**				
Current	75.8 (21.9)	76.0 (23.3)	78.3 (19.7)	74.1 (19.4)
Lifetime	107.9 (19.3)	109.6 (19.9)	104.7 (17.6)	104.9 (18.5)
Total	182.1 (39.5)	183.1 (42.0)	182.9 (35.3)	178.9 (35.1)
	*n* (%)	*n* (%)	*n* (%)	*n* (%)
**Age at Time of Trauma**				
Childhood	21 (7.8)	13 (11.1)	0	4 (10.5)
Adulthood	99 (36.5)	44 (37.6)	5 (29.4)	18 (47.4)
Both	151 (55.7)	60 (51.3)	12 (70.6)	16 (42.1)
**Type of Trauma**				
Sexual	145 (53.5)	69 (59.0)	8 (47.1)	19 (50.0)
Combat	6 (2.2)	1 (0.9)	0	2 (5.3)
Other	4 (1.5)	1 (0.9)	1 (5.9)	0
Combination (sexual + )	116 (42.8)	46 (39.3)	8 (47.1)	17 (44.7)
**No. of Traumas**				
>One	222 (81.9)	96 (82.1)	13 (76.5)	25 (65.8)
**PTSD Diagnosis**				
PTSD Plus	183 (67.5)	78 (66.7)	9 (52.9)	27 (71.1)
**Ethnicity**				
Non-Hispanic White	165 (60.9)	75 (64.1)	7 (41.2)	24 (63.2)
Hispanic	69 (25.5)	26 (22.2)	6 (35.3)	10 (26.3)
African American	23 (8.5)	10 (8.6)	1 (5.9)	2 (5.3)
Native American	12 (4.4)	6 (5.1)	3 (17.7)	2 (5.3)
Other	2 (0.7)	0	0	0
**Marital Status**				
Married	79 (29.2)	35 (29.9)	5 (29.4)	12 (31.6)
Divorced	110 (40.6)	45 (38.5)	6 (35.3)	19 (50.0)
Never Married	76 (28.0)	37 (31.6)	6 (35.3)	6 (15.8)
Widowed	6 (2.2)	0	0	1 (2.6)

Note: CAPS = Clinician Administered PTSD Scale for DSM-IV; *n* = number of participants, *M* = mean, *SD* = standard deviation, Ind = Individuals. † = Completer participants; ‡ = number of groups.

### 2.2. Measures

The initial assessment consisted of a semi-structured psychosocial interview, demographics, and interview administration of the Clinician Administered PTSD Scale (CAPS) [20] to determine PTSD diagnosis. The CAPS, based on the Diagnostic and Statistical Manual of Mental Disorders (DSM-IV) [20], is a structured interview administered by a trained clinician and is considered the gold standard in diagnosing PTSD. The 17 PTSD symptoms were assessed for frequency and intensity in the past month and lifetime. The CAPS has shown internal consistency for the three symptom categories of PTSD—reexperiencing, avoidance/numbing, and hyperarousal–with alpha coefficients which have ranged from 0.73 to 0.85; and good convergent validity between the CAPS and other measures of PTSD [21]. Internal consistency using Cronbach’s alpha was computed on the 17 CAPS symptom scores in the clinic sample and revealed an overall alpha of 0.85 with item correlations > 0.40 for all symptom items except psychogenic amnesia (symptom 8), which had a correlation of 0.12. 

The PTSD Symptom Checklist (PCL) [22] was administered at the first and last group session to document pre/post PTSD treatment changes. The PCL is a 17-item, five-point Likert, self-report scale with PTSD symptoms anchored from 1 (not at all) to 5 (extremely). The PCL was scored as follows to meet DSM-IV symptom criteria: 1) minimum of 1 reexperiencing, 3 avoidance/numbing, and 2 hyperarousal symptoms rated moderately (3) or higher; and 2) total symptom severity ≥ 50. The PCL is frequently used in clinical settings and can be used as a continuous variable or to dichotomize groups by presence or absence of diagnostic symptoms. The measure has a high correlation (*r* = 0.93) with the CAPS, high internal consistency (alpha = 0.94), with a sensitivity of 0.78, specificity of 0.86, and diagnostic efficiency of 0.83 [23]. 

### 2.3. Procedures

Three cognitive treatment protocols were delivered in a group format in 8-, 10-, and 12-session-lengths as the first treatment in a larger program followed by other group interventions (support, assertiveness and relaxation training, exposure therapies; [24]). The 8-session protocol was a structured manualized cognitive therapy [25] protocol with the same principles/goals as CPT of teaching the cognitive restructuring of maladaptive beliefs in the five domains from CPT [1] and were conducted between 1995 and 2007.The 10- and 12-session CPT-C (excluded exposure component) protocols [12] were conducted from 2008 to 2013. In the 12-session CPT-C protocol, the two trauma writing/narrative review sessions were replaced with two additional sessions where thoughts and feelings were identified [12]. All groups followed structured, manualized protocols and were delivered by trained (by the first author and/or certified in CPT), licensed clinicians. Trained psychology interns and post-doctoral fellows co-facilitated group sessions. Fidelity was not systematically monitored. 

### 2.4. Data Analysis

Descriptive statistics (means [*M*], standard deviations [*SD*], and correlations) were used to present demographics. Clinical differences were assessed with Chi-square tests for categorical variables and analysis of variance (ANOVA) for continuous variables, with Fisher’s Least Significance Difference method for post hoc comparisons of means; and *p*-values ≤ 0.05 were considered statistically significant. Pre/post was modeled as a repeated factor in a repeated measures (RM) ANOVA analysis.

An intention-to-treat (ITT) analysis was first conducted on the total sample (*n* = 271), with the last observation carried forward (LOCF) for participants missing post-treatment PCL scores, defined as non-completers. The data for completers (*n* = 172), defined as participants with attendance in the first and last group session and with pre/post-treatment PCL scores, was then analyzed. Paired *t*-tests were computed on pre/post PCL values, first on the total sample and then for completers. Changes in PTSD for the different group lengths were compared with an ANOVA. Next, the data were re-analyzed with an ANOVA using group as the unit of analysis (*n* = 47) for mean pre/post PCL scores to control for within-group correlations and resulting inflation of type I error [26].

## 3. Results

An ITT paired *t*-test on pre/post PCL scores was conducted on the full sample (*N* = 271) across all groups (*n* = 51), imputing LOCF for missing data, and significant decreases in PTSD were found (*p* < 0.001), with a medium effect size (*ES* = 0.38; see Table 2). Having established that PTSD significantly decreased with the conservative ITT, the remaining analyses were conducted on completer participants. In the completer sample (*n* = 172 individuals), there were significant pre/post treatment decreases on total PCL scores (*p* < 0.001, *ES* = 0.54), as well as in the symptom categories of reexperiencing (*p* < 0.001, *ES* = 0.37), avoidance/numbing (*p* < 0.001, *ES* = 0.53), and hyperarousal (*p* < 0.001, *ES* = 0.47; see Table 2) with medium effect sizes. These results suggest cognitive treatment delivered in a group setting reduces PTSD. In order to control variance contributed to the findings from intraclass correlation, the completer sample data were re-analyzed using the group as the unit of analysis (*n* = 47 groups). Significant pre/post treatment decreases were found, with large effect sizes for total PCL (*p* < 0.001, *ES* = 0.92) and for the symptom categories of reexperiencing (*p* < 0.001, *ES* = 0.77), avoidance/numbing (*p* < 0.001, *ES* = 0.90), and hyperarousal (*p* < 0.001, *ES* = 0.71; see Table 2).

**Table 2:** PCL means, standard deviations, and effect sizes for cognitive group participants.

	Total Sample (*n* = 271)		Completers (*n* = 172)		Group as Unit (*n* = 47)	
	*M (SD)*	*ES*	*M (SD)*	*ES*	*M (SD)*	*ES*
**Total PCL Score**						
Pre	65.0 (11.3)		65.0 (10.9)		65.0 (6.5)	
Post	61.4 (13.7) ^a^	0.38	58.4 (14.7) ^a^	0.54	58.7 (8.4) ^a^	0.92
**Reexperiencing Symptoms**						
Pre	18.6 (4.1)		18.6 (4.1)		18.6 (2.4)	
Post	17.8 (4.7) ^a^	0.27	17.0 (5.0) ^a^	0.37	17.1 (2.8) ^a^	0.77
**Avoidance/Numbing Symptoms**						
Pre	26.6 (5.3)		26.5 (5.1)		26.6 (2.8)	
Post	24.9 (6.2) ^a^	0.37	23.4 (6.5) ^a^	0.53	23.6 (3.7) ^a^	0.90
**Hyperarousal Symptoms**						
Pre	19.7 (3.7)		19.8 (3.6)		19.8 (2.4)	
Post	18.7 (4.2) ^a^	0.33	17.9 (4.7) ^a^	0.47	18.0 (2.7) ^a^	0.71

Note: ^a ^*p* < 0.001; PCL = PTSD Symptom Checklist; *M* = Mean, *SD* = Standard Deviation, *ES* = Effect Size.

The next step was to examine the impact of group length on treatment outcome in the completer sample. The 8-session group contained 32 groups, the 10-session group 5 groups and the 12-session group 9 groups. The mean number and range of participants along with demographics is presented in Table 1. No significant differences in baseline demographics were found between participants in the three group lengths. The impact of group length on PTSD improvement was examined through the change in PCL scores among completers in the 8-, 10-, and 12-session groups employing a 3 × 2 (8/10/12 × pre/post) RMANOVA. Neither a significant interaction (*p* = 0.68) nor a significant main effect for group length (*p* = 0.32) was found. However, a significant main effect for treatment was found (*p* < 0.001), with post hoc testing showing significant PCL improvement in all three groups (all *p* < 0.001). The greatest improvement was found in the 10-session group (8 session *M* = 5.7; 10 session *M* = 9.1; 12 session *M* = 7.9; see Table 3); however, these improvements were not significantly different (*p* = 0.06). Finally, dose of therapy was tested by examining percent of sessions attended. The percent of attendance was significantly different (*p* < 0.001) for each group length, with the 8-session group showing significantly higher attendance (86.8%) than the other two group lengths (10-session = 76.5%, 12-session = 72.6%). An ANOVA was conducted on PCL improvement, overall attendance, and group length. A significant main effect was found for attendance (*p* = 0.04) in PCL score improvement, however interactions were not found, suggesting PTSD improvement did not vary by group length and attendance is of greater importance than group length. 

**Table 3:** Means and standard deviations for PCL improvement and group length.

	Pre PCL Score	Post PCL Score	Effect Size		Percent Improvement
	*M (SD)*	*M (SD)*		*p* value	*M (SD)*
**Group Length**					
8-Session (*N* = 117)	65.2 (11.6)	59.5 (15.4)	.43	< 0.001	8.3 (9.4)
10-Session (*N* = 17)	65.6 (10.2)	56.5 (15.4)	1.15	< 0.001	14.8 (12.9)
12-Session (*N* = 38)	63.8 (9.3)	55.9 (12.0)	.60	< 0.001	11.5 (19.3)

Note:^ a ^*p* < 0.001; PCL = PTSD Symptom Checklist; *M* = Means; *SD* = Standard Deviations.

Finally, clinical improvement in PTSD symptoms [27] was examined to capture: 1) response to treatment (PCL improvement ≥ 10 and ≥ 20 points; [28]); 2) loss of diagnosis (PCL < 50 and fewer than 1 reexperiencing, 3 avoidance/numbing, and 2 hyperarousal symptoms; [20]); and 3) complete remission (PCL < 35; [29]). Subjects subclinical for PTSD (*n* = 20) at baseline were eliminated from this analysis and included 14 from the 8-session group, 2 from the 10-session group, and 4 from the 12-session group. In the remaining combined sample, 30% dropped 10 or more PCL points; 12% dropped 20 or more PCL points, 22% lost the PTSD diagnosis; and 8% were in complete remission (see Table 4). In the 10-session group, the largest percent of participants showed a 10-point decrease (41%) and loss of diagnosis (33%) than the other two groups.

**Table 4:** PCL clinical improvement and group length for completer subjects.

	10+ Point Decrease	20+ Point Decrease	Loss of Diagnosis	Complete Remission
	*n (%)*	*n (%)*	*n (%)*	*n (%)*
**Group Length**				
8-Session (*N* = 103)	33 (28.2)	10 (8.6)	20 (19.4)	12 (10.3)
10-Session (*N* = 15)	7 (41.2)	2 (11.8)	5 (33.3)	0 (0.0)
12-Session (*N* = 34)	12 (31.6)	8 (21.1)	9 (26.5)	1 (2.6)
All Groups Combined (*N* = 152)	52 (30.2)	20 (11.6)	34 (22.4)	13 (7.6)

Note: PCL = PTSD Symptom Checklist; *N* = total number of participants; *n* = number of participants in group; Loss of Diagnosis = PCL total score < 50 and not meeting DSM-IV criteria (< 1 reexperiencing symptom, < 3 avoidance numbing symptoms, and < 2 hyperarousal symptoms). Complete remission = PCL total score of < 35.

## 4. Discussion

Our study demonstrates improvement in PTSD symptoms using a group format for CPT-C cognitive restructuring models with differing session lengths in an applied clinical setting with women veterans, supporting our first hypothesis. As in individually delivered CPT-C [8,11] and other individual cognitive therapy models [2], PTSD symptoms improved after group treatment in our study using three analytic approaches to demonstrate the veracity of the results. The approaches included a conservative ITT analysis, the individual as the unit of analysis in the completer sample, and using group as the unit of analysis. The ITT analysis controlled for bias in outcome due to dropout subjects. Analysis of completer subjects provided results on subjects receiving treatment. Finally, examination of the data using group as the unit of analysis controlled for intraclass correlation and violation of assumption of independence in statistical analyses, both of which have been criticized in group treatment studies in the past [26], and result in inflation of Type I error. The compelling results of the statistical analyses are supported by a number of clinical improvement indicators. Clinical PTSD improvement was demonstrated by response to treatment, with a third of the sample showing 10 or more PCL point decreases and 20% no longer meeting diagnostic criteria for PTSD. The overall outcome results support group-delivered CPT-C for the treatment of PTSD.

Our second hypothesis that more treatment sessions would produce greater reductions in symptoms was not confirmed. While all three group lengths—8, 10, or 12 sessions—produced significant decreases in PTSD, differences between the group lengths were not found. The direction of change was also unexpected with the largest for 10-session, over the 8- and 12-session groups. The small number of subjects in the 10-session group (*n* = 17) likely contributed to insufficient power to detect statistical differences. It should be noted that while higher attendance was found in the 8-session group subjects, the 10-session group subjects showed greater PTSD improvement, suggesting a 10-session group was long enough to consolidate change. Additionally, a higher response to treatment and loss of diagnosis was found in the 10-session group length. While session length differences were not statistically found in our study, length of treatment remains an important consideration; given higher dropout rates were associated with the longer treatment (12-session) in our study, as has been found in others [30].

Although the present study provides notable contributions to the small body of literature examining group-delivered PTSD treatments, these findings should be considered in light of limitations. First, the failure to find significant differences among the three groups was likely due to a lack of power, given the small number of participants in the 10-session length group. Other limitations are typical in the examination of clinical samples and include the lack of randomization, reliance on a self-report outcome measure, and treatment adherence not assessed to assure treatment integrity. Finally, while our overall cognitive group treatment results can be generalized to other PTSD populations (with ITT), analyses on the completer participants may be limited to older females with fewer traumatic experiences, as they were significantly different from the non-completers.

## 5. Conclusions

Our results support and enhance the literature examining a group format for cognitive treatments for PTSD by demonstrating cognitive therapy can be an effective time-limited treatment. From an economic standpoint, the group format provides the advantage of efficiency over individual therapy, improving access to care for PTSD while decreasing costs. While our study was not sufficiently powered to detect an optimal length for group-delivered cognitive treatment, minimum number of sessions for cognitive group therapy can be addressed in future studies. Future research should also focus on examining the impact of group-only CPT within other clinical populations and/or settings, utilizing random assignment, and including an active control condition. Although the current study was not without its limitations, it supports the implementation of future randomized control trails of group CPT with other clinical populations. 
